# Metabolic syndrome risk in adult coffee drinkers with the rs301 variant of the LPL gene

**DOI:** 10.1186/s12937-024-00931-7

**Published:** 2024-03-02

**Authors:** Pao-Chun Hsieh, Oswald Ndi Nfor, Chuan-Chao Lin, Chih-Hsuan Hsiao, Yung-Po Liaw

**Affiliations:** 1 Department of Obstetrics and Gynecology, Chung-Kang Branch, Cheng Ching Hospital, Taichung City, 407 Taiwan; 2 Department of Public Health, Institute of Public Health, Chung Shan Medical University, Taichung, 40201 Taiwan; 3 Department of Physical Medicine and Rehabilitation, Chung Shan Medical University Hospital, Taichung, Taiwan; 4 School of Medicine, Chung Shan Medical University, Taichung City, 40201 Taiwan; 5 Institute of Medicine, Chung Shan Medical University, Taichung City, 40201 Taiwan; 6 Department of Medical Imaging, Chung Shan Medical University Hospital, No. 110 Sec. 1 Jianguo N. Road, Taichung City, 40201 Taiwan

**Keywords:** Metabolic syndrome, Polymorphism, Coffee

## Abstract

**Background:**

Metabolic syndrome (MetS), a cluster of metabolic and cardiovascular risk factors is influenced by environmental, lifestyle, and genetic factors. We explored whether coffee consumption and the rs301 variant of the lipoprotein lipase (LPL) gene are related to MetS.

**Methods:**

We conducted multiple logistic regression analyses using data gathered from 9523 subjects in Taiwan Biobank (TWB).

**Results:**

Our findings indicated that individuals who consumed coffee had a reduced odds ratio (OR) for MetS (0.750 (95% confidence interval [CI] 0.653–0.861) compared to non-coffee drinkers. Additionally, the risk of MetS was lower for individuals with the ‘TC’ and ‘CC’ genotypes of rs301 compared to those with the ‘TT’ genotype. Specifically, the OR for MetS was 0.827 (95% CI 0.721–0.949) for the ‘TC’ genotype and 0.848 (95% CI 0.610–1.177) for the ‘CC’ genotype. We observed an interaction between coffee consumption and the rs301 variant, with a p-value for the interaction of 0.0437. Compared to the reference group (‘no coffee drinking/TT’), the ORs for MetS were 0.836 (95% CI 0.706–0.992) for ‘coffee drinking/TT’, 0.557 (95% CI 0.438–0.707) for ‘coffee drinking/TC’, and 0.544 (95% CI 0.319–0.927) for ‘coffee drinking/CC’. Notably, MetS was not observed in non-coffee drinkers regardless of their rs301 genotype.

**Conclusion:**

Our findings suggest that rs301 genotypes may protect against MetS in Taiwanese adults who consume coffee compared to non-coffee drinkers.

## Introduction

Metabolic syndrome is a severe condition characterized by the presence of at least 3 of the following components: central adiposity, elevated fasting glucose, blood pressure, triglycerides (TGs), and low plasma levels of high-density lipoprotein (HDL) cholesterol [1–3]. It is a global health concern associated with an increased risk type 2 diabetes, cardiovascular disease, and other chronic diseases [4]. Moreover, this disorder is becoming increasingly prevalent, especially among older adults in Taiwan [5]. Previously, we found that sex and age interact to affect this condition [6]. According to a previous study [7], Taiwanese women are more prone to developing this condition than men (31.5% vs. 25.5%).

Notably, the prevalence of this multifactorial disorder is increasing worldwide. Genetics, lifestyle, and environmental factors are essential to understanding the interactions between the various components associated with MetS [8]. Coffee, one of the most-consumed beverages in the world, is among lifestyle factors believed to improve MetS [9–11] possibly due to its antioxidant and anti-inflammatory properties [12]. Moderate coffee drinking is beneficial to cardiovascular health [13].

Metabolic risk components are widely recognized as being inherited and several genetic variants have already been investigated for MetS. One gene that has been implicated in metabolic syndrome is the LPL gene, which encodes for lipoprotein lipase, an enzyme involved in the metabolism of lipids. A specific variant of this gene, rs301 has been associated with metabolic syndrome and its components in diverse populations [14, 15]. Notably, this association has not been extensively replicated in Asians.

It has been suggested that individual genetic profiles might influence how the body metabolizes coffee [16]. Despite this, the complete understanding of the effects of coffee consumption on metabolic syndrome and its underlying genetic factors remains to be determined. In Taiwan, there is a dearth of studies investigating the interplay between genetic variants, coffee consumption, and the risk of MetS. To address this gap, we utilized electronic data from the TWB to examine the interactive influence of coffee and LPL rs301 on the risk of developing MetS.

## Materials and methods

### Data source and study population

In the current study, we analyzed TWB data for 9523 subjects assessed at recruitment centers across Taiwan between 2008 and 2016. Before the assessment, participants had provided written informed consent. Following the completion of the questionnaires, they were examined physically and blood samples were collected for DNA analysis. Of the overall subjects enrolled in the current study, we excluded 1662 subjects with missing data (i.e., those with incomplete questionnaires and missing genotype data). The final analysis involved 7861 eligible subjects. None of these subjects had a cancer diagnosis in the past. We obtained approval for this study from the institutional review board of Cheng Ching Hospital (HP200012).

### Study variables

Subjects were identified as having metabolic syndrome if they displayed a minimum of 3 of the following characteristics: waist circumference of ≥ 90 cm (35 inches) for men or ≥ 80 cm (31 inches) for women, systolic blood pressure ≥ 130mmHg and diastolic blood pressure ≥ 85mmHg, or were actively on antihypertensive medication, fasting blood glucose ≥ 100 mg/dL, or were receiving treatment for diabetes, fasting triglyceride ≥ 150 mg/dL or were currently receiving treatment for hypertriglyceridemia, and HDL-C < 40 mg/dL for men or < 50 mg/dL for women. These criteria are based on the scientific statement on MS diagnosis issued by the American Heart Association and National Heart, Lung, and Blood Institute [17].

Lifestyle data were gathered through questionnaires administered to each participant at the time of initial assessment at each center. These factors, which have also been explored previously for metabolic syndrome [18–20] included smoking, physical activity, diet (vegetarian diet, non-vegetarian diet, and midnight snacking), and alcohol consumption. The questionnaire included the frequency and type of coffee consumed, such as coffee with creamer, plain black coffee, or coffee with milk, as well as the approximate volume consumed. Subjects were categorized as regular coffee consumers if they had ingested a minimum of one cup of coffee (approximately ≥ 130 ml/cup) per day, at least three days per week, over the past six months, and were still consuming coffee during the time of evaluation. Smoking and alcohol intake were categorized as never, current and former smokers, and drinkers. Exercise was defined as having any form of physical activity at least three times and over 30 min each time in a week.

### Genotyping

Using search engines like Science Direct, PubMed, Snpedia, and Google Scholar, we chose variant rs301 of the LPL gene. Because of its association with metabolic syndrome and its components, it was deemed appropriate to include it in the study. Details on sample collection and genotyping are available on the TWB website [21]. As part of the quality control, the call rate was > 95%. The minor allele frequency (MAF) was > 0.05 while the *p*-value for the Hardy-Weinberg equilibrium (HWE) test was > 0.001.

### Statistical analysis

We employed SAS software (version 9.4, SAS Institute, Cary, NC, USA) and PLINK v1.90 for our data analyses. We stratified baseline variables based on coffee consumption and presented data as numbers and percentages. Categorical variables were compared using the Chi-square (χ2) test, and we determined genotypic associations using multiple logistic regression analysis.

## Results

The baseline characteristics of participants are presented in Table 1. Metabolic syndrome was identified in about 16.2% (*n* = 427) of those who drank coffee. Female coffee drinkers were more than male drinkers (55.14% vs. 44.86%). Of the overall samples, the OR for MetS in coffee drinkers compared with nondrinkers was 0.750 (95% CI 0.653–0.861) as shown in Table 2. Compared with ‘TT’ of rs301, the OR for MetS was 0.827 (95% CI 0.721–0.949) and 0.848 (95% CI 0.610–1.177) for ‘TC and CC,’ respectively. The corresponding ORs were 1.825 (95% CI 1.507–2.209), 3.123 (95% CI 2.582–3.778), and 4.464 (95% CI 3.603–5.529) for the 41 to 50, 51 to 60, and 61 to 70 compared to the 30 to 40 year age group. Overweight, obese, and smoking individuals had ORs of 3.943 (95% CI 3.323–4.678), 14.600 (95% CI 12.266–17.378), and 1.564 (95% CI 1.275–1.917), respectively. There was an interaction between coffee drinking and rs301 (*p* for interaction = 0.0437). After stratification (Tables 3 and 4), coffee drinkers carrying ‘TT, TC, and CC’ genotypes had ORs of 0.852 (95% CI 0.718–1.011), 0.599 (95% CI 0.463–0.774), and 0.477 (95% CI 0.229–0.992), respectively. With the no coffee drinking and ‘TT’ group used as the reference group, significant ORs were 0.836 (95% CI 0.706–0.992) for ‘coffee drinking/TT,’ 0.557 (95% CI 0.438–0.707) for coffee with ‘TC,’ and 0.544 (95% CI 0.319–0.927) for ‘coffee drinking/CC,’ respectively (Table 4). MetS was not observed among non-coffee drinkers, no matter the rs301 genotype.

**Table 1:** Baseline characteristics of the study subjects

	No coffee consumption	Coffee consumption	p-value
	(*n* = 5226)	(*n* = 2635)	
**Metabolic Syndrome**			< 0.001
No	4165(79.70)	2208(83.80)	
Yes	1061(20.30)	427(16.20)	
**rs301**			0.009
TT	3306(63.26)	1671(63.42)	
TC	1737(33.24)	836(31.73)	
CC	183(3.50)	128(4.86)	
**Sex**			< 0.001
Female	2619(50.11)	1453(55.14)	
Male	2607(49.89)	1182(44.86)	
**Age (year)**			< 0.001
30 to 40	1414(27.06)	785(29.79)	
41 to 50	1430(27.36)	849(32.22)	
51 to 60	1499(28.68)	669(25.39)	
61 to 70	883(16.90)	332(12.60)	
**BMI (kg/m** ^**2**^ **)**			0.176
Normal	2554(48.87)	1255(47.63)	
Overweight	1577(30.18)	849(32.22)	
Obese	1095(20.95)	531(20.15)	
**Smoking**			0.001
Never	4064(77.77)	1946(73.85)	
Former	591(11.31)	340(12.90)	
Current	571(10.93)	349(13.24)	
**Alcohol intake**			0.392
Never	4693(89.80)	2347(89.07)	
Former	155(2.97)	75(2.85)	
Current	378(7.23)	213(8.08)	
**Exercise**			0.002
No	3348(64.06)	1691(64.17)	
Aerobic	1687(32.28)	805(30.55)	
Non aerobic	191(3.65)	139(5.28)	
**Midnight snacking**			0.072
No	3609(69.06)	1767(67.06)	
Yes	1617(30.94)	868(32.94)	
**Vegetarian diet**			< 0.001
No	4692(89.78)	2426(92.07)	
Former	276(5.28)	82(3.11)	
Current	258(4.94)	127(4.82)	

Data were presented as numbers and percentages.

Abbreviation: MetS = Metabolic Syndrome, BMI = body mass index; TT, TC, and CC are the genotypes of the variant rs301

**Table 2:** The odds of metabolic syndrome among study subjects

	OR	95% CI	*p*-value
**Coffee drinking (ref**: No**)**			
Yes	0.750	0.653–0.861	< 0.001
**rs301 (ref**: TT**)**			
TC	0.827	0.721–0.949	0.007
CC	0.848	0.610–1.177	0.325
**Sex (ref**: Female**)**			
Male	0.855	0.737–0.992	0.039
**Age**, year **(ref**: 30 to 40**)**			
41 to 50	1.825	1.507–2.209	< 0.001
51 to 60	3.123	2.582–3.778	< 0.001
61 to 70	4.464	3.603–5.529	< 0.001
**BMI (kg/m**^**2**^**) (ref**: Normal**)**			
Overweight	3.943	3.323–4.678	< 0.001
Obese	14.600	12.266–17.378	< 0.001
**Smoking (ref**: Never**)**			
Former	0.931	0.758–1.143	0.494
Current	1.564	1.275–1.917	< 0.001
**Alcohol intake (ref**: Never**)**			
Former	1.420	1.025–1.964	0.034
Current	1.257	0.997–1.586	0.053
**Exercise (ref**: No**)**			
Aerobic	0.852	0.738–0.983	0.028
Non-Aerobic	0.787	0.564–1.099	0.159
**Midnight snacking (ref**: No**)**			
Yes	1.176	1.024–1.350	0.022
**Vegetarian diet (ref**: No**)**			
Former	1.166	0.851–1.596	0.339
Current	0.974	0.722–1.315	0.866

Abbreviation: OR = odds ratio; ref = reference; BMI = body mass index; TT, TC, and CC are the genotypes of the variant rs301.

**Table 3:** Risk of metabolic syndrome among subjects based on stratification by genotypes of the rs301 variant

	TT			TC			CC		
	OR	95% CI	p-value	OR	95% CI	p-value	OR	95% CI	p-value
**Coffee drinking (ref**: No**)**									
Yes	0.852	0.718–1.011	0.067	0.599	0.463–0.774	< 0.001	0.477	0.229–0.992	0.047
**Sex (ref**: Female**)**									
Male	0.941	0.782–1.132	0.517	0.738	0.564–0.965	0.027	0.447	0.187–1.069	0.070
**Age (ref**: 30 to 40**)**									
41 to 50	1.814	1.431–2.299	< 0.001	2.051	1.441–2.920	< 0.001	0.661	0.240–1.821	0.423
51 to 60	3.133	2.471–3.972	< 0.001	3.507	2.477–4.966	< 0.001	1.731	0.701–4.277	0.234
61 to 70	4.650	3.558–6.077	< 0.001	4.711	3.186–6.967	< 0.001	2.551	0.887–7.332	0.082
**BMI (kg/m**^**2**^**) (ref**: Normal**)**									
Overweight	3.647	2.954–4.503	< 0.001	4.560	3.335–6.233	< 0.001	5.279	2.077–13.415	0.001
Obese	14.989	12.097–18.571	< 0.001	14.116	10.231–19.478	< 0.001	22.899	8.576–61.141	< 0.001
**Smoking (ref**: Never**)**									
Former	0.766	0.591–0.992	0.043	1.325	0.925–1.898	0.125	1.192	0.334–4.261	0.787
Current	1.333	1.031–1.725	0.029	2.040	1.424–2.923	< 0.001	4.609	1.538–13.813	0.006
**Alcohol intake (ref**: Never**)**									
Former	1.962	1.319–2.919	0.001	0.906	0.482–1.696	0.757	0.137	0.020–0.937	0.043
Current	1.193	0.886–1.607	0.246	1.492	1.001–2.222	0.049	0.659	0.206–2.109	0.482
**Exercise (ref**: No**)**									
Aerobic	0.858	0.719–1.025	0.091	0.790	0.609–1.024	0.075	1.099	0.506–2.386	0.811
Non-Aerobic	0.777	0.511–1.181	0.237	0.761	0.419–1.383	0.370	0.582	0.103–3.281	0.540
**Midnight snacking (ref**: No**)**									
Yes	1.192	1.004–1.415	0.045	1.087	0.846–1.395	0.514	1.571	0.716–3.448	0.260
**Vegetarian diet (ref**: No**)**									
Former	1.398	0.947–2.066	0.092	0.943	0.542–1.644	0.837	0.274	0.035–2.125	0.216
Current	1.042	0.716–1.516	0.830	0.972	0.572–1.652	0.917	0.280	0.041–1.896	0.192

Abbreviation: OR = odds ratio; ref = reference; BMI = body mass index; TT, TC, and CC are the genotypes of the variant rs301

**Table 4:** Risk of metabolic syndrome based on coffee consumption and genotypes of rs301

	OR	95% CI	p-value
**Coffee drinking and rs301 genotype (ref: No coffee drinking/TT)**			
No coffee drinking/TC	0.903	0.768–1.063	0.222
No coffee drinking/CC	0.986	0.653–1.488	0.945
Coffee drinking/TT	0.836	0.706–0.991	0.039
Coffee drinking/TC	0.557	0.438–0.707	< 0.001
Coffee drinking/CC	0.544	0.319–0.927	0.025

Abbreviation: OR = odds ratio, ref = reference; TT, TC, and CC are the genotypes of the variant rs301

Adjusted for sex, age, BMI, smoking, alcohol consumption, exercise, midnight snacking, and vegetarian diet

## Discussion

In our comprehensive examination involving 7861 subjects from the TWB, we uncovered intriguing associations between coffee consumption, the rs301 polymorphism of the LPL gene, and MetS. Notably, our investigation delved into the interplay between genetic predisposition and lifestyle choices in the context of MetS risk. We identified a protective effect of coffee consumption against MetS, a finding reinforced by the significant odds ratios associated with ‘TT, TC, and CC’ genotypes of the rs301 polymorphism.

Results from an earlier cross-sectional study implied that consuming a moderate amount of coffee, ranging from 1 to 4 cups per day, could enhance metabolic health [11]. However, a different study suggested that this positive relationship is only evident with substantial coffee intake [22]. In contrast, a study involving 1040 Finnish populations found that higher consumption coffee consumption was linked to an increased risk of MetS [23]. Our research challenges the previously reported heightened risk associated with increased coffee consumption, proposing that regular coffee intake may, in fact, decrease the risk of MetS. The specific mechanisms driving these observed associations are not yet fully understood, but it seems that polyphenols in coffee may play a role [24, 25].

Recent reports from the Jakarta Post and an earlier publication [26] indicate a growing coffee culture on the island. Due to the potential association of coffee consumption with unhealthy behaviors, it had been suggested that individual genetic profiles be analyzed in coffee-related studies [16]. In our exploration of the connection between coffee intake, the rs301 variant, and MetS, we observed that individuals with ‘TT, TC, and CC’ genotypes were less prone to developing metabolic syndrome among Taiwanese coffee consumers. Although non-coffee drinkers exhibited lower odds ratios, these differences were not statistically significant.

Additionally, we identified positive associations between older age, overweight, obesity, and smoking with MetS. Obesity is a commonly observed component of MetS [27] even though weak associations have been documented [28]. Current smoking remained a risk factor for MetS regardless of the rs301 genotype, aligning with existing evidence supporting the connection between active smoking and an elevated risk of MetS [29–31]. Age is a well-known risk factor for MetS. After stratification, we found that the age-dependent risk of MetS significant primarily among carriers of the ‘TT’ and ‘TC’ genotypes.

Our study’s findings have certain implications for both public health policies and individual lifestyle decisions, particularly within populations exhibiting a high prevalence of the rs301 variant. The study suggests that individuals who consume coffee have a lower odds ratio for MetS compared to non-coffee drinkers. This finding aligns with existing research indicating potential health benefits associated with moderate coffee consumption, such as improved metabolic health and reduced cardiovascular risk [32]. An interesting observation is the interaction between coffee consumption and the rs301 variant. We found a significant p-value for interaction, indicating that the protective effect of the rs301 genotypes against MetS was more pronounced among coffee drinkers. This interaction underscores the importance of considering both genetic factors and lifestyle choices in understanding metabolic health. Given that the study was conducted in Taiwanese adults, the findings may have cultural and regional implications. Therefore, public health initiatives and communication strategies may need to be adapted to suit the preferences and dietary habits of specific populations.

While our study boasts a sizable sample size, the limitation includes a lack of detailed information on caffeine intake and coffee preparation methods. Further research, encompassing diverse populations and exploring the molecular mechanisms underlying these associations, is warranted to validate and expand upon our findings.

## Conclusions

In summary, the study suggests that the rs301 genotype may confer protection against MetS, particularly in the context of coffee consumption, and more so in older adults. These findings have the potential to inform personalized health strategies and contribute to the development of targeted interventions for metabolic health within specific populations.

## Data Availability

The data that support the findings of this study are available from Taiwan Biobank but restrictions apply to the availability of these data, which were used under license for the current study, and so are not publicly available. Data are however available from the authors upon reasonable request and with permission of Taiwan Biobank.

## Abbreviations

MetS: metabolic syndrome
LPL: lipoprotein lipase
TWB: Taiwan Biobank
OR: odds ratio
CI: confidence interval
